# Supplementary material for: Live streaming mode selection strategy under the background of virtual anchor supplementation
Source: PLoS One. 2025 Apr 16;20(4):e0321557. doi: 10.1371/journal.pone.0321557 (PMC12002449; doi:10.1371/journal.pone.0321557)
Supplement: Attachment — Submitted filename: rebuttal letter_1.docx [file pone.0321557.s003.docx]

Dear Editor and Reviewers,

Thanks very much for taking your time to review this manuscript. We really appreciate all your comments and suggestions. In accordance with the instructions in your letter, we have uploaded the revised version of the manuscript with all the changes highlighted by using the track changes mode in MS Word. Appended to this letter is our itemized response to the comments raised by the reviewer. The comments from reviewers are reproduced in italic type and our responses are given directly afterward in a different color (red). Thanks again!

**Reviewer #1**

*Q1: The author's modifications to the title did not reach a satisfactory level, and even the revised title became ambiguous.*

**Answer1:** Thank you for your suggestions, we have simplified the title of the paper and now modify it to:

Live streaming mode selection strategy under the background of virtual anchor supplementation.

*Q2: The author's answer to question 2 is too simple and does not present the motivation of this study logically and clearly. I suggest the author to reorganize the* *entire introduction section.*

**Answer2:** We thank the reviewer for pointing this out. We have reorganized the content of the entire introduction section, and the relevant content is as follows (Lines 45-64 on page 3; Lines 65-85 on page 4; Lines 86-92 on page 5):

Live streaming is becoming increasingly popular worldwide ^[1]^. In daily life, both businesses and consumers can sell and purchase products on platforms such as Instagram Live Shopping, Twitter's e-commerce module, Taobao Live, Facebook Live, and Amazon Live ^[2]^. With the development of live streaming e-commerce ^[3]^, new live streaming modes are constantly emerging. Businesses can choose suitable live streaming modes based on factors such as live streaming anchor type, live streaming anchor characteristics, and consumer preferences ^[4,5,6,7]^, such as merchant live streaming mode, influencer anchor live streaming mode, etc. With the development of artificial intelligence technology, using virtual anchors as a supplement to human anchors has become a new hybrid live streaming mode. For example, well-known beauty brands such as Carslan, L'Oreal, and Perfect Diary choose merchant staff anchors or influencer anchors such as Viya and Li Jiaqi for live streaming sales. After midnight, they also use virtual anchors such as "Big Eye Kaka", "Ou Xiaomi", and "Stella" to replace human anchors and continue selling products.

The mixed live streaming mode of human anchors and virtual anchors can use virtual anchors to fill the time gap when human anchors cannot live streaming sales. However, the construction and application of virtual anchors require a certain cost investment. At present, the low-end version of virtual anchors on the market is priced at tens of thousands of yuan. Merchants can achieve "7 * 24" hour live streaming sales of virtual anchors by purchasing anchor software and operation and maintenance services. The high-end version of virtual anchors is mostly personalized customization, with different levels of pricing based on the difficulty of customization, with an average price of around 200000 yuan. Due to the poor live streaming interaction ability of virtual anchors, most live streaming rooms choose to use a combination of "human anchors and virtual anchors" for live streaming sales, manufacturers need to pay for the construction and application costs of virtual anchors in addition to the cost of human anchors. Therefore, based on cost and profit considerations, whether manufacturers choose traditional live streaming modes such as merchant live streaming mode and influencer anchor live streaming mode, or choose to invest in and apply virtual anchors on the basis of the traditional live streaming mode to obtain additional income, is a question worth studying.

Based on this, in the context of virtual anchors as a supplement to human anchors in live streaming e-commerce. In this paper, the first time to include virtual anchors in the live streaming mode selection strategy, combined with the characteristics of the live streaming anchor and the cost of live streaming, four live streaming modes are constructed: merchant live streaming mode, influencer anchor live streaming mode, merchant combined with virtual anchors live streaming mode, and influencer anchor combined with virtual anchors live streaming mode. Explore the optimization problem of manufacturer's live streaming mode selection decision. The problems to be solved are as follows:

(1) What are the optimal live streaming channel prices and manufacturers' optimal profits under different live streaming modes?

(2) What impact do parameters such as cross price elasticity coefficient, market share of live streaming channels and consumer sensitivity to live streaming e-commerce have on live streaming channel prices?

(3) What is the manufacturer's live streaming mode selection strategy considering the impact of parameters such as cross price elasticity coefficient, market share of live streaming channels and consumer sensitivity to live streaming e-commerce?

*Q3: I think the author's explanation of question 3 is unreasonable or the author did not answer this question. I suggest the author to explain it from both theoretical and practical perspectives.*

**Answer3:** Thank you for your suggestions. We have made corrections to the issue of viewing e-tailers as Stackelberg leaders, as follows (Lines 236-254 on page 12):

In the context of virtual anchors as a supplement to human anchors in live streaming e-commerce, when manufacturers choose to expand their sales channels through live streaming sales, there are four modes: merchant live streaming mode, influencer anchor live streaming mode, merchant combined with virtual anchors live streaming mode, and influencer anchor combined with virtual anchors live streaming mode. In the merchant live streaming mode and the merchant combined with virtual anchors live streaming mode, manufacturers play a dominant role in the game with e-tailers based on their strong financial strength and long-term product development ^[35,36,37]^. Therefore, when manufacturers establish live streaming channels to sell products, they will prioritize determining the live streaming channel price, and e-tailers will determine the e-tailing channel price based on the manufacturer's live streaming pricing; In the influencer anchor live streaming mode and the influencer anchor combined with virtual anchors live streaming mode, manufacturers hire influencer anchors for live streaming sales, such as well-known influencer anchors with a large number of fans such as Li Jiaqi, Viya, Dong Yuhui, etc. Due to their influence, they have certain pricing power in live streaming sales ^[38,39]^. Therefore, when manufacturers establish live streaming channels to sell products, influencer anchors will first determine the live streaming channel price, and e-tailers will determine the e-tailing channel price based on the live streaming pricing of influencer anchors.

In addition, based on the latest game sequence, we have also made relevant modifications to the model results and simulation analysis. Among them, the model results have changed slightly, while the content of simulation analysis has not changed much. Some modifications are as follows (Lines 316-331 on page 16; Lines 332-345 on page 17), and all modifications have been reflected in the main text (Lines 236-254 on page 12; Lines 316-331 on page 16; Lines 332-345 on page 17; Lines 348-350 on page 17; Lines 351-367 on page 18; Lines 368-381 on page 19; Lines 384-399 on page 20; Lines 400-412 on page 21; Lines 416-432 on page 22; Lines 433-446 on page 23; Lines 447-455 on page 24; Lines 456-473 on page 25; Lines 474-493 on page 26; Lines 494-505 on page 27).

The profit function of the e-tailer and the manufacturer are as follows:

$$\pi_{1E}=\left(p_{1e}-w\right)D_{1e}=\left(p_{1e}-w\right)\left(a-ak-p_{1e}+rp_{1l}\right)$$

The first-order derivative of the e-tailer's profit function $\pi_{1E}$ concerning the e-tailing channel price $p_{1e}$ is:

$$\frac{\partial\pi_{1E}}{\partial p_{1e}}=w-2p_{1e}+rp_{1l}-ak+a$$

From equation (11), we can see that $\frac{\partial\pi_{1E}^{2}}{\partial p_{1e}^{2}}=-2<0$；the e-tailer's profit function is a concave function of the price of the e-tailing channel, so there is a maximum value of the e-tailer's profit function. To equation (11) for the first-order derivative condition on $p_{1e}$ can be obtained under the e-tailer's profit maximization of the optimal price solution, so that $\frac{\partial\pi_{1E}}{\partial p_{1e}}=0$, can be obtained e-tailing channel price equilibrium solution:

$$p_{1e}=\frac{rp_{1l}+w-ak+a}{2}$$

The first order derivative of the manufacturer's profit function $\pi_{1M}$ with respect to the live streaming channel price $p_{1l}$ is:

From equation (13) , we can see that $\frac{\partial\pi_{1M}^{2}}{\partial p_{1l}^{2}}=r^{2}-2<0,\left(0<r<0.5\right)$；The manufacturer's profit function $\pi_{1M}$ is a concave function with respect to the price of the live streaming channel $p_{1l}$, hence there exists a maximum value of the manufacturer's profit function $\pi_{1M}$.

Substituting Eq. (11) into Eq. (12) and taking the first-order derivative of the live streaming channel price, the live streaming channel price equilibrium solution can be obtained:

Substituting Eq. (14) into Eq. (11), the e-tailing channel price equilibrium is solved as:

In price equilibrium, the e-tailer's profit and the manufacturer's profit are:

References:

35. Pu X, Sun S, Shao J. Direct selling, reselling, or agency selling? Manufacturer’s online distribution strategies and their impact. International Journal of Electronic Commerce. 2020, 24(2): 232-254.

36. Shi G, Jiang L, Wang Y. Interaction between the introduction strategy of the third‐party online channel and the choice of online sales format. International Transactions in Operational Research. 2022, 29(4): 2448-2493.

37. Wang T Y, Chen Z S, Govindan K, et al. Manufacturer’s selling mode choice in a platform-oriented dual channel supply chain. Expert Systems with Applications. 2022, 198: 116842.

38. Yang L, Zheng C, Hao C. Optimal platform sales mode in live streaming commerce supply chains. Electronic Commerce Research. 2024, 24(2): 1017-1070.

39. Du S, Dong Q, Peng J, et al. Brand or regular manufacturer? Influencer's cooperation strategies under reputation risk. IEEE Transactions on Engineering Management. 2023.
