# Supplementary material for: Live streaming mode selection strategy under the background of virtual anchor supplementation
Source: PLoS One. 2025 Apr 16;20(4):e0321557. doi: 10.1371/journal.pone.0321557 (PMC12002449; doi:10.1371/journal.pone.0321557)
Supplement: Attachment — Submitted filename: rebuttal letter.docx [file pone.0321557.s002.docx]

Dear Editor and Reviewers,

Thanks very much for taking your time to review this manuscript. We really appreciate all your comments and suggestions. In accordance with the instructions in your letter, we have uploaded the revised version of the manuscript with all the changes highlighted by using the track changes mode in MS Word. Appended to this letter is our itemized response to the comments raised by two reviewers. The comments from reviewers are reproduced in italic type and our responses are given directly afterward in a different color (red). Thanks again!

**Reviewer #1**

*Q1: The title of the paper is not concise enough.*

**Answer1:** Thank you for your suggestions, we have simplified the title of the paper and now modify it to:

Strategies for pricing and mode selection for manufacturer live streaming with virtual anchor supplementation.

*Q2: In the introduction section, the motivation of this paper should be explained clearly.*

**Answer2:** We thank the reviewer for pointing this out. Our explanation of the motivation for writing the paper in the introduction section is supplemented as follows (Lines 56-62 on page 3):

In the context of the virtual anchors as a supplement to human anchors in live streaming e-commerce, the merchant combined with virtual anchors live streaming mode, influencer anchor combined with virtual anchors live streaming mode, while playing the advantages of human anchors and virtual anchors, but the virtual anchor live interaction ability is limited, and need to invest a certain cost, after the introduction of virtual anchors, how the price of live streaming should be set, and how to choose between different live streaming modes remains to be studied. (Lines 56-62 on page 3).

*Q3: The author needs to further explain why the e-retailer is the Stackelberg leader rather than the influencer anchor or the manufacturer?*

**Answer3:** Thank you for your suggestions, Our explanation regarding e-tailer as the leader of Stackelberg is as follows(Lines 239-250 on page 12):

When a manufacturer establishes a live streaming channel, it will seize the market share of the existing e-tailers, and there is a certain competitive relationship between the manufacturer and the e-tailers. Compared with the e-tailers, the manufacturer is in a low competitive position due to the fact that the online consumer group it has accumulated and cultivated is not yet in scale. The influencer anchors are an option for manufacturers, and they are in a short-term employment relationship, leaving out the participation of influencer anchors in gaming for the time being. Therefore, this paper assumes that the Stackelberg game is played between the manufacturer and the e-tailer, with the e-tailer as the dominant player and the manufacturer as the follower, the e-tailer first determines the price of the e-tailing channel, and then the manufacturer sets the price of the live streaming channel, and then the consumer finally decides which sales channel to buy the product from. (Lines 239-250 on page 12).

*Q4: I don't quite understand the situation where the influencer anchor live streaming selling combined with the virtual anchor mode, are there any real-life cases to support this?*

**Answer4:** Thank you for your suggestions, Our supplementary explanation on the influencer anchor combined with virtual anchors for internet celebrities is as follows(Lines 50-55 on page 3)：

For example, Kazilan, Perrier, perfect diary and other well-known beauty brands, choose merchants staff anchor or Viya, Li Jiaqi and other influencer anchor live sales at the same time, after 12 midnight, will also use “big eyes Kaka” and other virtual anchor instead of the human anchor to continue to sell the product, to fill the live streaming time gap, this creates a new space for revenue growth. (Lines 50-55 on page 3).

*Q5:  I don't quite understand why the author defines the number of anchors t and why t<30?*

**Answer5:** Thank you for your suggestions, Our supplementary explanation on the influencer anchor combined with virtual anchors for internet celebrities is as follows：

Thank you for your suggestions, the main purpose of setting the number of live streaming of human anchors in the model is to indicate that virtual anchors have the advantage of not being limited by time and continuously broadcasting to increase product exposure compared to human streamers. After considering that the Number of live streaming of human anchors cannot effectively demonstrate the impact of continuous streaming by virtual anchors on increasing product exposure，and the continuous live streaming of virtual anchors, due to their novelty[1,2] and the exposure effect, will to some extent affect consumers' willingness to purchase[3]. so replace the Number of live streaming of human anchors $t$ with Level of investment in continuous live streaming for virtual anchors, Level of investment in continuous live streaming for virtual anchors indicates the advantage of virtual anchors not being limited by time and increasing product exposure through continuous live streaming. The specific changes to the variables and model related parts are described below.

The demand functions for the merchant combined with virtual anchors, and the influencer anchor combined with virtual anchors, have been adjusted accordingly (Lines 295-306 on page 15). The specific changes are as follows:

The demand functions for the e-tailing channel and the live streaming channel in the merchant combined with virtual anchors live streaming mode are:

$$D_{3r}=\left(1-k\right)a-p_{3r}+rp_{3m}$$

Among them, the exposure effects of virtual anchor input application levels $\sigma(0<\sigma<1)$ indicates the extent of the application of virtual host for continuous live streaming, which will indirectly increase the exposure of the live room in the live streaming platform, which in turn affects the consumer's willingness to buy.

The demand functions for the e-tailing channel and the live-streaming channel in the influencer anchor combined with virtual anchors live streaming mode are:

$$D_{4r}=\left(1-k\right)a-p_{4r}+rp_{4m}+\eta h$$

The profit function for the merchant combined with virtual anchors, and the influencer anchor combined with virtual anchors, have been adjusted accordingly (Lines 371-373 on page 20; Lines 402-404 on page 22). The specific changes are as follows:

In the merchant combined with virtual anchors, the profit function between the e-tailer and the manufacturer is:

$$\pi_{3r}=\left(p_{3r}-w\right)D_{3r}=\left(p_{3r}-w\right)\left(a\left(1-k\right)-p_{3r}+rp_{3m}\right)$$

In influencer anchor combined with virtual anchors, the profit function between the e-tailer and the manufacturer is:

$$\pi_{4r}=\left(p_{4r}-w\right)D_{4r}=\left(p_{4r}-w\right)\left(a\left(1-k\right)-p_{4r}+rp_{4m}+\eta h\right)$$

References

1.	Gao JY, Zhao XJ, Zhai MF, Zhang D, Li G. AI or Human? The Effect of Streamer Types on Consumer Purchase Intention in Live Streaming. International Journal of Human-Computer Interaction. 2024.

2.	Zhang YX, Wang XY, Zhao X. Supervising or assisting? The influence of virtual anchor driven by AI-human collaboration on customer engagement in live streaming e-commerce. Electronic Commerce Research. 2023.

3. Xie K , Lee Y J .Social Media and Brand Purchase: Quantifying the Effects of Exposures to Earned and Owned Social Media Activities in a Two-Stage Decision Making Model.Journal of Management Information Systems. 2015; 32(2):204-238.

*Q6: In the analysis section, the authors' explanation of the proposition focuses more on explaining the results and lacks exploration of the underlying reasons. The explanation of propositions is more about explaining why such results are produced, which factors or effects play an important role in it, so as to provide a basis for subsequent management insights.*

**Answer6:** Thank you for your suggestions, We provide additional explanations on the innovative points of our research in this article，Here are some of the contents（Lines 514-530 on page 30；Lines 531-542 on page 31）.with all changes reflected in the main text（Lines 550-552 on page 31；Lines 553-573 on page 32；Lines 574 on page 33；Lines 583-595 on page 33；Lines 596-610 on page 34）.

As shown in Fig3, the manufacturer's profit is directly proportional to the cross price elasticity coefficient. When the cross price elasticity coefficient changes, the profit changes of the four live streaming modes are analyzed and expressed as follows. Firstly, as the cross price elasticity coefficient increases, the profit of the merchant combined with virtual anchors live streaming mode (the influencer anchor combined with virtual anchors live streaming mode) is higher than that of the merchant live streaming mode (the influencer anchor live streaming mode). When the cross price elasticity coefficient increases, the change in demand for products in online retail channels is more easily affected by the price of live streaming products. The higher the substitutability of products in live streaming channels to products in online retail channels, the more manufacturers can supplement live streaming sales with virtual anchors, increase live streaming exposure, and make more consumers aware of live streaming products, thereby increasing live streaming sales revenue. Secondly, when the cross price elasticity coefficient is low, the influencer anchor live streaming mode is the optimal choice. When the cross price elasticity coefficient is low, the substitutability of products from live streaming channels to products from online retail channels is small, and products from live streaming channels do not have product advantages. At this time, merchant staff anchors do not have enough attractiveness to consumers, while influencer anchors can attract more consumer attention and purchase products due to their personal influence. Hiring influencer anchors for live sales can bring more income. Thirdly, when the cross price elasticity coefficient is high, the merchant combined with virtual anchors live streaming mode is the optimal choice. When the cross price elasticity coefficient is large, the substitutability of products from live streaming channels to products from online retail channels is higher, and products from live streaming channels have a certain competitive advantage. Compared with the higher commission rate of influencer anchors, the live streaming cost of merchant staff anchors is lower, and manufacturers gain more profits. Therefore, when the cross price elasticity coefficient is large, merchant combined with virtual anchors live streaming mode is the optimal choice.（Lines 514-530 on page 30；Lines 531-542 on page 31）.

*Q7: The innovation of this paper is not clear. There are many existing literature studies on the live streaming e-commerce. So, the specific contributions and innovations of this article in terms of research perspective, research conclusions, etc. need to be clearly explained.*

**Answer7:** We thank the reviewer for pointing this out. The specific supplementary explanation for the innovative points of this study is as follows (Lines 197-208 on page 10):

Different from previous studies, this paper, in the context of virtual anchors as a supplement to human anchors in live streaming e-commerce, considers the introduction of virtual anchors from merchant live streaming mode, influencer anchor live streaming mode, respectively, and constructs four different live streaming modes, which enriches the live streaming mode model. Secondly, for the first time, virtual anchors are included in the live streaming pricing and mode selection decisions, and the optimal live streaming channel pricing of manufacturers is studied by synthesizing live streaming anchor characteristics and live streaming costs. Finally, based on the impact of parameters such as cross price elasticity coefficient, market share of live streaming channels and consumer sensitivity to live streaming e-commerce on manufacturers' profits, it provides a reference for manufacturer's live streaming mode selection decisions. (Lines 197-208 on page 10).

*Q8: The research conclusions of this article are relatively intuitive, and no innovative conclusions have been obtained from the study.*

**Answer8:** Thank you for your suggestions, We have further supplemented the lack of innovation in our research conclusions，Here are some of the contents（Lines 665-677 on page 37）.with all changes reflected in the main text（Lines 650-658 on page 36；Lines 659-679 on page 37; Lines 680-700 on page 37）:

When the cross price elasticity coefficient increases, the higher the substitutability of products in live streaming channels to products in online retail channels, the manufacturers can supplement live streaming sales with virtual anchors, increase live streaming exposure, and make more consumers aware of live streaming products, thereby increasing live streaming sales revenue. Secondly, when the cross price elasticity coefficient is low, the influencer anchor live streaming mode is the optimal choice. The substitutability of products from live streaming channels to products from online retail channels is small, hiring influencer anchors for live sales can bring more income. Thirdly, when the cross price elasticity coefficient is high, the substitutability of products from live streaming channels to products from online retail channels is higher. Compared with the higher commission rate of influencer anchors, the live streaming cost of merchant staff anchors is lower, merchant combined with virtual anchors live streaming mode is the optimal choice. （Lines 665-677 on page 37）.

*Q9: The language is not smooth enough, it is recommended to polish the overall language of the paper.*

**Answer9:** Thank you for your suggestions, we have revised and polished the overall language of the paper.

**Reviewer #2**

*Q1: In the Abstract, add a clear statement of the paper's unique contribution, e.g., "This study is the first to incorporate virtual anchors into live streaming mode selection strategies."*

**Answer1:** We thank the reviewer for pointing this out. Our explanation of the unique contribution of this article in the abstract is as follows（Lines 2-6 on page 1）:

In the context of virtual anchors as a supplement to human anchors in live streaming e-commerce. In this paper, for the first time, virtual anchors are included in the live streaming pricing and mode selection strategy, combined with the characteristics of the live streaming anchor and the cost of live streaming, constructed a model of the four live streaming modes, and used the Stackelberg game method to study.（Lines 2-6 on page 1）.

*Q2.1: In lines 41-42, include global examples beyond Taobao Live, Facebook Live, and Amazon Live. Consider mentioning platforms like Instagram Live Shopping or Twitter's "Shop Module".*

**Answer2.1:** Thank you for your suggestions, we have supplemented the examples of Instagram Live Shopping or Twitter's "shopping module" in the introduction, We have supplemented the examples of Instagram Live Shopping or Twitter's "shopping module" in the introduction, the specific supplementary content is as follows （Lines 24-28 on page 2）:

Live streaming is becoming increasingly popular globally ^[1]^, with both merchants and consumers selling and buying products on platforms such as Instagram Live Shopping, Twitter's e-commerce module, Taobao Live, Facebook Live, and Amazon Live in their daily lives ^[2]^, which has given rise to a new form of business called live streaming commerce ^[3]^.（Lines 24-28 on page 2）.

*Q2.2: After line 57, add a paragraph summarizing key literature on live streaming e-commerce strategies to establish the research context.*

**Answer2.2:** We thank the reviewer for pointing this out. A summary and explanation of key literature on live streaming e-commerce strategies, as follows (Lines 37-42 on page 2):

When merchants carry out live sales services, they need to determine the live streaming price and live streaming mode, current scholars from the live streaming anchor type, live streaming anchor characteristics, consumer preferences, consumer behavior and other perspectives, with the help of the Stackelberg game approach to research, for the manufacturer's live streaming pricing and mode selection to provide the relevant decision support. (Lines 37-42 on page 2).

*Q2.3: On line 77, provide a brief explanation of how the Stackelberg game method applies to this study.*

**Answer2.3:** We thank the reviewer for pointing this out. The supplementary explanation on the application of Stackelberg game method in this study is as follows (Lines 70-74 on page 4):

In addition, the live streaming channel, as a new sales channel, will seize the market share of the existing e-tailers, and there is a certain competitive relationship between manufacturers and e-tailers, so the Stackelberg game method is applied to study the live streaming pricing and mode selection problem of manufacturers. (Lines 70-74 on page 4).

*Q3.1: After the Introduction, add the following sections in order: Literature Review, Methodology, Results, Discussion, Conclusion.*

**Answer3.1:** Thank you for your suggestions. We have adjusted the structure order of this article according to the chapters of Introduction, Literature Review, Methodology, Results, Discussion, and Conclusion, and improved the content arrangement of the Methodology and Results chapters. At this point, the methodology chapter includes content such as problem description, model parameters, basic assumptions, and demand function models. The results section includes profit function models for online retailers and manufacturers under different live streaming modes, model solution results, and equilibrium solution analysis.

*Q3.2: At the end of the Introduction, add a paragraph outlining the paper's structure.*

**Answer3.2:** We thank the reviewer for pointing this out. We provide an overview of the structure of the paper in the introduction section, with the following specific additions (Lines 92-96 on page 5):

The rest of the paper is organized as follows: section 2, reviews the related literature. Section 3, performs problem description and constructs mathematical models. Section 4, solves and analyzes the equilibrium solution. In Section 5, analyzes the impact of parameters and discusses the limitations and future research. In Section 6, summarizes the main conclusions of this paper. (Lines 92-96 on page 5).

*Q4.1: After line 94, clearly define each of the four live streaming modes and explain how they were derived.*

**Answer4.1:** We thank the reviewer for pointing this out. Our definition and derivation of the four live streaming modes are supplemented as follows (Lines 62-64 on page 3; Lines 65-70 on page 4):

This paper considers the introduction of virtual anchors from the merchant live streaming mode, influencer anchor live streaming mode respectively, the live mode is divided into four: merchant live streaming mode which choose the company's staff for live streaming; influencer anchor live streaming mode which hire influencer anchors for live streaming; merchant combined with virtual anchors live streaming mode which choose the company's staff for live streaming while introducing virtual anchors; influencer anchor combined with virtual anchors live streaming mode which hire influencer anchors for live streaming while introducing virtual anchors. (Lines 62-64 on page 3; Lines 65-70 on page 4).

*Q4.2: Provide full mathematical formulations for the Stackelberg game model used in the study.*

**Answer4.2:** We thank the reviewer for pointing this out. We have fully supplemented the demand function formula, profit function formula, and mathematical formula used in the solution process of the Stackelberg game model in the paper, with all changes reflected in the main text (page14; page15; page16; page17; page18; page19; page20; page21; page22; page23; page24; page25).

*Q5.1: Create a table summarizing the optimal profits for e-tailers and manufacturers under each of the four live streaming modes.*

**Answer5.1:** We thank the reviewer for pointing this out. We are sorry to inform you that due to the long length of the complete mathematical formulas for the optimal profit of online retailers and manufacturers, which affects the layout of the article to some extent, a summary table of optimal profit has not been added at the relevant position in the article, we hope you can understand.

*Q5.2: Include graphs showing how key variables (e.g., price competition intensity, consumer sensitivity) affect mode selection.*

**Answer5.2:** We thank the reviewer for pointing this out. We have added content on how key variables affect the selection of live streaming modes in our analysis. The relevant parts are as follows (Lines 514-516 on page 30), with all changes reflected in the main text (Lines 514-530 on page 30; Lines 531-542 on page 31; Lines 550-552 on page 31; Lines 553-573 on page 32; Lines 574 on page 33; Lines 583-595 on page 33; Lines 596-610 on page 34):

As shown in Fig3, the manufacturer's profit is directly proportional to the cross price elasticity coefficient. When the cross price elasticity coefficient changes, the profit changes of the four live streaming modes are analyzed and expressed as follows. Firstly, as the cross price elasticity coefficient increases, the profit of the merchant combined with virtual anchors live streaming mode (the influencer anchor combined with virtual anchors live streaming mode) is higher than that of the merchant live streaming mode (the influencer anchor live streaming mode). When the cross price elasticity coefficient increases, the change in demand for products in online retail channels is more easily affected by the price of live streaming products. The higher the substitutability of products in live streaming channels to products in online retail channels, the more manufacturers can supplement live streaming sales with virtual anchors, increase live streaming exposure, and make more consumers aware of live streaming products, thereby increasing live streaming sales revenue. (Lines 514-516 on page 30).

*Q6: After presenting the results, add a section comparing your findings with those of previous studies, such as the work mentioned in lines 64-67 about Gree Electric Chairman Dong Mingzhu's live streaming success.*

**Answer6:** We thank the reviewer for pointing this out. We have made relevant comparisons based on Xin et al.'s research on live streaming mode selection, and the specific content is as follows (Lines 613-616 on page 34; Lines 617-635 on page 35)：

Xin et al. discuss brand self-live streaming, influencer-led live streaming mixture, and influencer-led special live streaming in terms of the anchor's live streaming service level and consumers' sensitivity to live streaming, and find that influencer-led special live streaming is superior to brand self-live streaming as consumers' sensitivity to live streaming increases. In this paper, in the context of virtual anchors as a supplement to human anchors in live streaming e-commerce, the study finds that consumers' sensitivity to live sales sensitivity on the choice of live streaming mode, there are different findings. Firstly, when the consumer sensitivity to live streaming e-commerce is large, the profit of the merchant combined with virtual anchors live streaming mode gradually exceeds that of influencer anchor live streaming mode. This is because as consumers become more sensitive to live streaming sales, they are more likely to generate shopping desire under the influence of live streaming sales performance such as product introductions and live streaming interactions. At this time, using virtual anchors to supplement live streaming sales of human anchors can leverage the exposure effect of continuous live streaming by virtual anchors. Compared with influencer anchors, the live streaming cost of merchant staff anchors is lower. Therefore, the merchant combined with virtual anchors live streaming mode will gradually be better than the influencer anchor live streaming mode. Secondly, when the consumer sensitivity to live streaming e-commerce is large, the influencer anchor combined with virtual anchors live streaming mode is the optimal live streaming mode, relying on the personal influence of influencer anchors and the continuous exposure of virtual anchors, will attract more consumers' attention and purchase of products, becoming the best choice for manufacturers. (Lines 613-616 on page 34; Lines 617-635 on page 35).

*Q7:* *Add a new section before the Conclusion discussing at least three limitations of the current study and suggesting corresponding future research directions.*

**Answer7:** We thank the reviewer for pointing this out. Our supplementary explanation regarding limitations and future research is as follows (Lines 638-657 on page 36):

This paper has some limitations that can be expanded in future research. Firstly, consumers are influenced by live anchors to produce impulse buying behavior, which can easily lead to the return of live products, after which the issue of live mode selection can be studied by combining the expected regret and return rate of consumers; secondly, live channels can be broadly divided into content platforms represented by TikTok and e-commerce platforms represented by Taobao, and the issue of live mode selection can be studied by combining the operation mechanism and preferential subsidy mechanism of different live platforms; furthermore, e-tailing channels generally exist in two ways, resale by e-tailers and sales by e-commerce platforms, and the choice of resale and agency sales can be studied later. (Lines 638-657 on page 36).

*Q8.1:* *Throughout the paper, ensure consistent use of terms. For example, decide between "live streaming selling" and "live e-commerce" and use the chosen term consistently.*

**Answer8.1:** Thank you for your suggestions. We have made the necessary revisions to ensure consistency in the terminology used in the paper, and will now use the term 'live streaming e-commerce' uniformly.

*Q8.2:* *In lines 20-22, revise the sentence structure for clarity: "This paper, in the context of virtual anchors supplementing human anchors in live streaming e-commerce, combines the characteristics of live streaming anchors and the number of human anchor live streams to construct a model of four live streaming modes.".*

**Answer8.2:** Thank you for your suggestions. We would like to add the following specific modifications to the sentence structure (Lines 2-6 on page 1):

In the context of virtual anchors as a supplement to human anchors in live streaming e-commerce. In this paper, for the first time, virtual anchors are included in the live streaming pricing and mode selection strategy, combined with the characteristics of the live streaming anchor and the cost of live streaming, constructed a model of the four live streaming modes, and used the Stackelberg game method to study. (Lines 2-6 on page 1).

*Q9:* *In the Conclusion, add a paragraph specifically addressing how manufacturers can use the study's findings to select the most appropriate live streaming mode based on their specific circumstances (e.g., market share, available resources).*

**Answer9:** We thank the reviewer for pointing this out. We have added management insights in the conclusion on how manufacturers can choose the most suitable live streaming mode based on their specific situation. The relevant parts are as follows (Lines 665-677 on page 37), with all changes reflected in the main text (Lines 658 on page 36; Lines 659-679 on page 37; Lines 680-700 on page 38):

When the cross price elasticity coefficient increases, the higher the substitutability of products in live streaming channels to products in online retail channels, the manufacturers can supplement live streaming sales with virtual anchors, increase live streaming exposure, and make more consumers aware of live streaming products, thereby increasing live streaming sales revenue. Secondly, when the cross price elasticity coefficient is low, the influencer anchor live streaming mode is the optimal choice. The substitutability of products from live streaming channels to products from online retail channels is small, hiring influencer anchors for live sales can bring more income. Thirdly, when the cross price elasticity coefficient is high, the substitutability of products from live streaming channels to products from online retail channels is higher. Compared with the higher commission rate of influencer anchors, the live streaming cost of merchant staff anchors is lower, merchant combined with virtual anchors live streaming mode is the optimal choice. (Lines 665-677 on page 37).
