# Supplementary material for: Live streaming mode selection strategy under the background of virtual anchor supplementation
Source: PLoS One. 2025 Apr 16;20(4):e0321557. doi: 10.1371/journal.pone.0321557 (PMC12002449; doi:10.1371/journal.pone.0321557)
Supplement: Attachment — Submitted filename: Response to Reviewers.docx [file pone.0321557.s004.docx]

Dear Editor and Reviewers,

Thanks very much for taking your time to review this manuscript. We really appreciate all your comments and suggestions. In accordance with the instructions in your letter, we have uploaded the revised version of the manuscript with all the changes highlighted by using the track changes mode in MS Word. Appended to this letter is our itemized response to the comments raised by the reviewer. The comments from reviewers are reproduced in italic type and our responses are given directly afterward in a different color (red). Thanks again!

**Reviewer #3**

*Q1: The figures used in the study require refinement, as they are currently unclear in the PDF file. Please* *enhance the resolution of the images.*

**Answer1:** Thank you for your suggestions, we have *enhanced the resolution of the images.*

*Q2: What role do consumer trust and engagement with virtual anchors play in influencing purchasing behavior during live-streaming events, and how can these factors be quantified within the model? Please explain.*

**Answer2:** We thank the reviewer for pointing this out. Our supplementary explanation of this issue is as follows (Lines 236-244 on page 12):

Virtual anchors are a new type of live streaming anchor ^[22]^, and consumers' trust in virtual anchors can enhance their willingness to purchase ^[35]^. In addition, consumers also have trust issues with the live streaming sales performance of ordinary live streaming anchors, influencer anchors, and other live streaming anchors ^[28]^. Therefore, for ease of analysis, this article does not separately consider consumers' trust in virtual anchors as an influencing factor, but uniformly regards consumers' trust and sensitivity to the characteristics of live streaming anchors (trustworthiness, live streaming interaction ability, etc.) as consumers' sensitivity to live streaming e-commerce ^[5,28]^, and uses to represent it.

References:

5. Pan R, Feng J, Zhao ZL. Fly with the wings of live-stream selling-Channel strategies with/without switching demand. Production and Operations Management. 2022;31(9):3387-99.

22. Gao W, Jiang N, Guo QQ. How do virtual streamers affect purchase intention in the live streaming context? A presence perspective. Journal of Retailing and Consumer Services. 2023;73.

28. Ladhari R, Massa E, Skandrani H. YouTube vloggers' popularity and influence: The roles of homophily, emotional attachment, and expertise. Journal of Retailing and Consumer Services. 2020;54.

35. Yuan, L., Dennis, A.R. Acting like humans? Anthropomorphism and consumer's willingness to pay in electronic commerce. Journal of Management Information Systems. 2019; 36: 450-477.
